# Rac1 and Cdc42 are regulators of HRas^V12^-transformation and angiogenic factors in human fibroblasts

**DOI:** 10.1186/1471-2407-10-13

**Published:** 2010-01-12

**Authors:** Daniel M Appledorn, Kim-Hien T Dao, Sandra O'Reilly, Veronica M Maher, J Justin McCormick

**Affiliations:** 1 Carcinogenesis Laboratory, Michigan State University, East Lansing, Michigan, 48824-1302, USA; 2 Cell and Molecular Biology Program, Michigan State University, East Lansing, Michigan, 48824-1302, USA; 3 Department of Microbiology & Molecular Genetics, Michigan State University, East Lansing, Michigan, 48824-1302, USA; 4 Department of Biochemistry & Molecular Biology, Michigan State University, East Lansing, Michigan, 48824-1302, USA; 5 Current address: Department of Medicine, Division Hematology-Oncology, Center for Hematologic Malignancies, Oregon Health and Science University, 3181 SW Sam Jackson Park Rd. UHN73C, Portland, OR, 97239, USA

## Abstract

**Background:**

The activities of Rac1 and Cdc42 are essential for HRas-induced transformation of rodent fibroblasts. What is more, expression of constitutively activated mutants of Rac1 and/or Cdc42 is sufficient for their malignant transformation. The role for these two Rho GTPases in HRas-mediated transformation of human fibroblasts has not been studied. Here we evaluated the contribution of Rac1 and Cdc42 to maintaining HRas-induced transformation of human fibroblasts, and determined the ability of constitutively activated mutants of Rac1 or Cdc42 to induce malignant transformation of a human fibroblast cell strain.

**Methods:**

Under the control of a tetracycline regulatable promoter, dominant negative mutants of Rac1 and Cdc42 were expressed in a human HRas-transformed, tumor derived fibroblast cell line. These cells were used to determine the roles of Rac1 and/or Cdc42 proteins in maintaining HRas-induced transformed phenotypes. Similarly, constitutively active mutants were expressed in a non-transformed human fibroblast cell strain to evaluate their potential to induce malignant transformation. Affymetrix GeneChip arrays were used for transcriptome analyses, and observed expression differences were subsequently validated using protein assays.

**Results:**

Expression of dominant negative Rac1 and/or Cdc42 significantly altered transformed phenotypes of HRas malignantly transformed human fibroblasts. In contrast, expression of constitutively active mutants of Rac1 or Cdc42 was not sufficient to induce malignant transformation. Microarray analysis revealed that the expression of 29 genes was dependent on Rac1 and Cdc42, many of which are known to play a role in cancer. The dependence of two such genes, *uPA *and *VEGF *was further validated in both normoxic and hypoxic conditions.

**Conclusion(s):**

The results presented here indicate that expression of both Rac1 and Cdc42 is necessary for maintaining several transformed phenotypes in oncogenic HRas transformed human cells, including their ability to form tumors in athymic mice. Our data also indicate that expression of either activated Rac1 or Cdc42 alone is not sufficient for malignant transformation of human fibroblasts, although each is required for specific transformed phenotypes. Furthermore, our study elucidates that the expression of several highly significant cancer related genes require the activities of Rac1 and/or Cdc42 which may also play a critical role in cellular transformation.

## Background

The Ras-family of guanosine triphosphatases (GTPases) regulates multiple cell processes, including cellular proliferation, differentiation, and actin-cytoskeletal organization. Altered expression or activation of *Ras *oncogenes has been found in ~30% of human cancers [1,2]. Acting as a molecular switch, Ras cycles between an inactive GDP-bound state and an active GTP-bound conformation. In its active form, Ras initiates mitogenic signals through various pathways, including the well-studied Raf-MEK-ERK1/2, PI3K/Akt, and RalGDS cascades (reviewed in [3]).

Two members of the Ras superfamily of small GTPases, namely Rac1 and Cdc42, were first investigated in Swiss-3T3 mouse fibroblasts and found to be regulators of the actin cytoskeleton [4-6]. In these reports, it was shown that Rac1 controlled lamellipodia and ruffling behavior, whereas Cdc42 affected the extension of filipodia. In addition to their role as cytoskeletal regulators, these small GTPases contribute to the regulation of several signal transduction proteins, including p21-activated kinase (PAK), p38/stress-activated protein kinases (SAPK), c-jun *N*-terminal kinases (JNK), nuclear factor κB (NFκB), and serum-responsive factor (SRF) [7].

The activities of Rac1 and Cdc42 are required for transformation of NIH3T3 mouse fibroblasts and Rat1 fibroblasts by expression of oncogenic Ras [8,9]. In most studies, constitutively-active (V12) mutants, or dominant-negative (N17) mutants of Rac1 and/or Cdc42 have been used to elucidate the respective unique roles each protein plays in oncogene transformation. For example, in NIH3T3 mouse fibroblasts, as well as Rat1 fibroblasts, Rac1^V12 ^expression confers growth factor independence, whereas Cdc42^V12 ^expression confers anchorage independent growth [8]. However, in Swiss-3T3 mouse fibroblasts, expression of either constitutively active protein results in growth factor independent proliferation [10]. This indicates that Rac1 and Cdc42 may have distinct roles in transformation depending on the cell line and/or species from which the cells are derived. However, in all three rodent fibroblast cell lines previously evaluated, it has been shown that expression of activated Rac1 or Cdc42 potentiated the ability for these cells to form sarcomas following their subcutaneous injection into athymic mice [8-10]. Although it is clear that Rac1 and Cdc42 play a role in HRas^V12^-induced transformation of rodent fibroblasts, HRas^V12^-induced transformation of human fibroblasts has been considered to be mechanistically distinct [11].

The present study was designed to determine whether the activity of Rac1, or Cdc42, or both, is required for HRas^V12^-induced transformation of human fibroblasts. Moreover, we sought to identify Rac1-mediated and/or Cdc42-mediated gene expression differences in the context of oncogenic HRas signalling. Our data confirm that activation of both Rac1 and Cdc42 is required for such HRas^V12^-induced transformation. In addition, using a genomic array approach, we identified 29 genes whose expression in HRas^V12^-transformed cells is regulated by Rac1 and/or Cdc42. Many of these genes, e.g. vascular endothelial growth factor (*VEGF*) and urokinase plasminogen activator (*uPA*), have been shown to play a significant role in cancer.

## Methods

### Cell strains and culture conditions

Unless otherwise indicated, the growth medium for the human foreskin-derived fibroblasts, i.e. MSU-1.1 and PH3MT strains and their derivatives, was Eagle's minimal essential medium supplemented with 0.2 mM L-aspartic acid, 0.2 mM L-serine, 1.0 mM sodium pyruvate, and 10% supplemented calf serum (SCS) (Hyclone Laboratories, Logan, UT). Penicillin, 100 units/ml, and streptomycin, 100 μg/ml were also included. All cell lines were cultured at 37°C in a humidified incubator containing 5% CO_2_, 95% ambient air.

To determine the genetic changes required for malignant transformation of human fibroblasts, McCormick, Maher, and their colleagues developed the MSU-1 lineage of human fibroblasts, beginning with finite life span skin fibroblasts derived from the foreskin of a normal neonate [12]. The parental cell strain in that lineage (designated MSU-1.1) is a chromosomally-stable, near-diploid, telomerase-positive, infinite life span cell strain that has been shown to be capable of being transformed into malignant cells by transfection of numerous oncogenes including HRas, NRas and KRas [13-17]. For example, MSU-1.1 cells transfected with an over-expressed oncogenic T24 HRas protein containing an activating V12 mutation (HRas^V12^) formed sarcomas when injected subcutaneously into athymic mice [15]. The malignant cell line, designated PH3MT, derived from such a tumor was used in the present study.

### Construction of human cell strains expressing dominant-negative mutant genes under the control of tetracycline

The pTet-tTAk and pTet-splice vectors were purchased from Invitrogen (Carlsbad, CA). This Tet-off system allows tetracycline-regulated expression of genes in mammalian cells. To facilitate selection, a pTet-tTAk vector expressing a gene coding for histidinol resistance was constructed and designated pTet-tTak^HisR^. A pTet-splice vector containing a gene coding for puromycin resistance was also constructed and designated pTet^PuroR^. We derived the Flag-Cdc42^N17 ^and myc-Rac1^N17 ^mutant cDNAs by PCR amplification using PFU polymerase (Stratagene, La Jolla, CA), and separately subcloned each into the pTet^PuroR ^vector. The cDNA templates used for PCR amplification of Cdc42^N17 ^and Rac1^N17 ^were kindly provided respectively by Dr. K. Gallo (Michigan State University) and Dr. G. Bokoch (Scripps Institute, La Jolla, CA). We verified the integrity of the resulting pTet-Flag-Cdc42^N17 ^and pTet-myc-Rac1^N17 ^vector constructs by automated DNA sequencing (Visible Genetics, Bayer, Toronto, ON, Canada). Lipofectamine (Invitrogen, Carlsbad, CA) was used to transfect the plasmids into human cells per manufacturers protocol.

PH3MT cells were transfected with the pTet-tTak^HisR ^vector, selected using histidinol (1 mM) (Sigma, St. Louis, MO), and designated PH3MT-tTak-C1. Such cells were subsequently transfected with the empty vector pTet^PuroR^, or with the pTet^PuroR ^vector containing myc-Rac1^N17 ^cDNA, or with the pTet^PuroR ^vector containing FLAG-Cdc42^N17 ^cDNA, and selected for resistance to puromycin (0.5 μg/ml) (Sigma, St. Louis, MO). A puromycin resistant strain resulting from transfection of the empty pTet-tTAk vector was also prepared and was designated PH3MT-VC-C2. Two independent clones that exhibited tetracycline-regulated expression of myc-Rac1^N17 ^were chosen for study and designated PH3MT-Rac1^N17 ^(-C1 or -C2). Two independent clones with regulatable FLAG-Cdc42^N17 ^expression were similarly selected and designated PH3MT-Cdc42^N17 ^(-C1, and C2). A sixth cell strain, designated PH3MT-Rac1^N17^/Cdc42^N17^, expressing both myc-Rac1^N17 ^and FLAG-Cdc42^N17 ^dominant-negative proteins was also isolated.

### Construction of cell strains expressing constitutively-activated mutants

To generate MSU-1.1 cell strains expressing GFP-Rac1^V12 ^or GFP-Cdc42^V12 ^fusion proteins, the GFP nucleotide sequence from the pCRUZ-GFP vector (Santa Cruz Biotechnology, Santa Cruz, CA) was isolated and ligated into the pcDNA6-V5-HisA vector (Invitrogen, Carlsbad, CA), which confers blasticidin resistance. Rac1^V12 ^and Cdc42^V12 ^cDNA template sequences were purchased from University of Missouri-Rolla Research Center (UMR) (Rolla, MO), PCR-amplified, and ligated downstream of the GFP nucleotide sequence to enable the transfectants to express N-terminally-labelled proteins. The resulting constructs were transfected into the parental, non-transformed MSU-1.1 cell strain, and selected for resistance to blasticidin (1 μg/ml) (Invitrogen, Carlsbad, CA) and screened for expression of the GFP-fusion protein by fluorescence microscopy. Identified clones were isolated, expanded, and screened by Western blotting for GFP-tagged Rac1^V12 ^or Cdc42^V12 ^protein expression.

### Cell lysates and Western blot analysis

Whole cell protein extracts were prepared using a lysis buffer consisting of 50 mM Tris-HCl, pH 7.2, 150 mM NaCl, 50 mM NaF, 0.5% NP-40, 1 mM Na_3_V0_4_, 200 mM benzamidine, 1 mM PMSF, 25 μg/ml aprotinin, and 25 μg/ml leupeptin. Total protein concentration was quantified using the Coomassie protein assay reagent (Pierce Biotechnology, Rockford, IL). Lysates were denatured in 5× Laemelli sample buffer, separated by either 10% or 12% SDS-PAGE, and transferred to PVDF membrane. The membrane was blocked for 2 hr with Tris-buffered saline containing 0.1% Tween-20 (TBST) and 5% (w/v) non-fat milk. For the majority of the studies, the membrane was probed overnight with the primary antibody at 4°C, then probed for 1 hr at room temperature with the appropriate horseradish peroxidase-linked secondary antibody (Sigma and Santa Cruz Biotechnology). Both antibodies were diluted in TBST containing 5% milk. The membrane was incubated with the Supersignal West Pico chemiluminescent horseradish peroxidase substrate (Pierce Biotechnology, Rockford, IL) and then exposed to film. The primary antibodies used were anti-FLAG, 1:1000 dilution (Sigma, St. Louis, MO), anti-myc 9E10, 1:500 dilution, and anti-GFP, 1:1000 dilution (Santa Cruz Biotechnology, Santa Cruz, CA).

### Focus reconstruction assay

The focus reconstruction assay was performed essentially as described [15]. Briefly, MSU-1.1 cells were plated at a density of 5 × 10^4 ^cells per 100-mm diameter dish containing growth medium supplemented with 0.5% SCS and 20 mM HEPES (pH 7.4) in the presence or absence of tetracycline. On the following day, the cells to be assayed for focus reconstruction were plated (100-200 cells per dish) in these same dishes. Cells received fresh medium every fourth day. After 3 weeks of growth, the cells were fixed with neutral buffered formalin, stained with methylene blue, and then examined for quality and quantity of foci.

### Assay for growth factor independence

Cells were plated in growth medium containing 10% SCS into a series of 60-mm diameter dishes at a density of 10^4 ^cells per dish and incubated for 48 hr. The cells were then washed twice and growth medium containing 0.5% SCS was added. The number of cells in three replicate dishes was counted at the indicated intervals using a Coulter counter. An equation derived from a best-fit exponential curve was used to determine the doubling time of cells in log-phase growth. This experiment was repeated three times.

### Assay for anchorage-independence

Cells were suspended in 0.33% top agarose, then plated into 60-mm-diameter culture dishes at a 5 × 10^3^ cells per dish, and then covered with 2 mL of growth medium. The growth medium was replaced weekly. After three weeks, the cells were fixed with 2.5% glutaraldehyde. This experiment was repeated three times.

### Assay for tumorigenicity

To test PH3MT derived cell strains, 10^6 ^cells were injected subcutaneously into the right and left flank of a series of athymic Balb/c mice. To turn off the dominant-negative form of the proteins, tetracycline was added to the drinking water of half of the mice, at a concentration of 1 mg/ml. To mask the flavor of tetracycline, 5% sucrose was added to the drinking water of all mice. Tumor measurements were made weekly, and mice were sacrificed when a tumor with a volume of 0.5 cm^3 ^developed on either flank. The tumorigenicity results were displayed in the form of Kaplan-Meier survival plots using MedCalc Version 8.2 http://www.medcalc.be. A log-rank test was used to determine statistical significance.

To determine whether the MSU-1.1 derivative cell strains expressing GFP-Rac^V12 ^or GFP-Cdc42^V12 ^were capable of forming tumors in athymic mice, a 1 cm^3 ^absorbable gelatin sponge (Gelfoam size 50, Pharmacia) was placed in each flank. One week later, 10^7 ^cells were injected directly into the sponges at each injection site. Such sponges were used to ensure that a high number of injected cells were retained at each injection site.

### Affymetrix GeneChip expression analysis

PolyA+RNA was extracted using the Micropure PolyA+RNA extraction kit according to the manufacturer's instructions (Ambion, Applied Biosystems, Austin, TX). To synthesize cRNA products from 3 μg of polyA+RNA, the procedures described in the Affymetrix expression analysis technical manual were followed. Personnel at the Genomics Technology Sequence Facility at Michigan State University carried out the hybridization to the Affymetrix HU95A human genome chip and probed, washed, and scanned the arrays as described in the Affymetrix manual. Such analysis was carried out in duplicate.

Total mRNA expression was calculated for each gene represented on the Affymetrix chip. Expression differences were calculated within each experiment as well as between experiments. This approach resulted in four separate data sets, i.e. both data sets collected from cells grown in the presence of tetracycline were compared to both data sets collected from cells grown in the absence of tetracycline. To ascertain differentially expressed genes, one-way parametric analysis of variance (ANOVA) tests were performed by using a Benjamini-Hochberg false discovery rate (BH FDR) for multiple testing correction, with a *P *value of 0.05 using MAS5.0 software. The significant changes in each of the four comparisons, as determined by the MAS5.0 software, were identified using Microsoft Access query tool.

### ELISA analyses

To detect levels of secreted uPA or VEGF protein from PH3MT derived cells, cells were plated in 100 mm-diameter culture dishes in growth medium containing tetracycline (1 mg/ml). After 24 hr, the medium was removed, the cells were washed twice, and tetracycline-free medium was added. Cells were incubated for another 24 hr to allow for expression of dominant-negative genes. The cells were then washed twice, and given medium lacking serum. After 24 hr the cells were stimulated with growth medium, cobalt chloride (CoCl_2_) (100 μM), deferoxamine (DFO) (100 μM) or were incubated in a hypoxic chamber (1% O_2_) for 24 hr. To determine the level of secreted VEGF and uPA protein from MSU-1.1 derivative cell strains, 1.5 × 10^5 ^cells were plated in a 100 mm-diameter tissue culture dish containing growth medium, and incubated for 24 hr. The cells were then washed twice with serum-free medium, and serum-starved for another 24 hr before being stimulated with growth medium containing 10% SCS. The media were collected, and centrifuged at 5000 × g for 5 min. To detect secreted VEGF protein, we carried out a sandwich ELISA using the human VEGF DuoSet (R&D Systems, Minneapolis, MN) ELISA kit following the manufacturer's protocol. To detect secreted levels of uPA protein, a similar sandwich ELISA was carried out using the uPA ELISA kit (Oncogene Science, Bayer, Cambridge, MA) according to the manufacturer's protocol.

For all ELISA analyses, after the removal of conditioned medium, whole cell protein extracts were made, quantified, and used to normalize the levels of uPA and VEGF protein detected by dividing the concentration of uPA or VEGF protein in the conditioned medium by the total amount of protein in the whole cell extract, expressed as percent of vector control, or as fold-change. Such experiments were repeated three times. Error bars indicate the standard deviation from the mean. To determine statistical significance, the student's two-tailed *t*-test was used.

## Results

### Role of Rac1 and Cdc42 proteins in HRasV12-transformation of human fibroblasts

**Figure 1:**
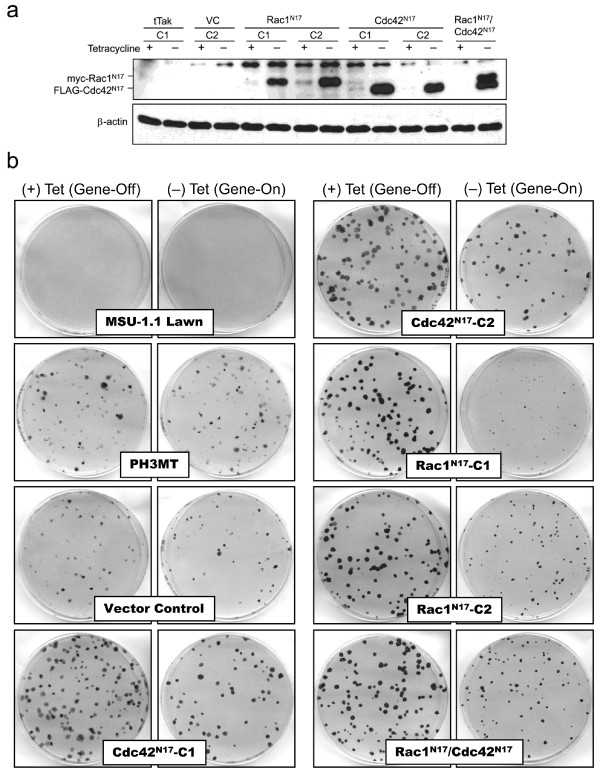
**A role for Rac1 and Cdc42 in focus reconstruction**. (a) Cells were grown in the presence or absence of tetracycline (1 μg/ml). PH3MT-tTak-C1 is the parental cell strain. PH3MT-VC-C2 is transfected with empty vector. Whole cell extracts were made and the Western blot was probed with both myc and FLAG antibodies. β-actin levels were used to indicate loading. (b) Cells expressing dominant negative mutants were plated on a lawn of non-transformed MSU-1.1 cells, and focus reconstruction was analyzed. Pictures are representative of replicate experiments.

To investigate whether the activity of Rac1, Cdc42, or both proteins, is required for HRas^V12^-induced malignant transformation of human fibroblasts, we transfected PH3MT cells, a cell line that had been malignantly-transformed by transfection of *HRas*^V12^, with plasmids encoding dominant negative forms of Rac1 (Rac1^N17^) and/or Cdc42 (Cdc42^N17^). It is possible that over-expression of these dominant negative proteins may alter other signalling pathways by sequestering exchange factors that are utilized by other members of the Rho GTPase family in addition to Rac1 and/or Cdc42. However, our use of these mutants allows for a direct comparison of studies completed in human fibroblasts presented here to those studies completed in rodent fibroblasts that used identical dominant-negative or constitutively-active mutants. In these cells, expression of dominant negative mutants is controlled in a Tet-off system, i.e., absence of tetracycline results in dominant negative protein expression. Regulated expression of dominant negative mutant proteins was observed in two independent clones of PH3MT cells transfected with plasmids containing either *Rac1*^N17 ^or *Cdc42*^N17 ^(Fig. 1a). Regulated expression of both Rac1^N17 ^and Cdc42^N17 ^was observed in one clone (Rac1^N17^/Cdc42^N17^). As expected, dominant negative mutant expression was not observed in parental cell strains PH3MT-tTak-C1 or a vector control transfected cell strain (PH3MT-VC-C2).

To evaluate the effect of dominant negative protein expression on the transformed phenotype of PH3MT cells, commonly used transformation assays including: growth curve analysis in reduced serum, focus reconstruction, and tumor forming ability, were carried out. Growth curve analysis revealed a significant increase in the doubling time in all dominant negative protein expressing clones cultured in medium lacking tetracycline (dominant negative protein expression) relative to their growth in medium containing tetracycline (dominant negative protein expression suppressed) (p < 0.05) (Table 1). A moderate increase in the doubling time of the vector control in the absence of tetracycline was also observed indicating some toxicity of tTak expression (p < 0.05). However, the extent to which tTak expression alone affected doubling time was significantly less than what was observed in all cell strains expressing dominant negative proteins (p < 0.01). Focus reconstruction assays also revealed a significant decrease in the ability of dominant negative protein expressing clones to form foci on a lawn of non-transformed human fibroblasts (MSU-1.1 cells) (Fig. 1b). In general, expression of Rac1^N17 ^affected both growth in reduced serum, and focus reconstruction properties to a greater extent than did expression of Cdc42^N17^. These results suggest that dominant negative protein expression does indeed affect the transformed properties of PH3MT cells, and suggest that the activities of both Rac1 and Cdc42 play a significant role in the ability of HRas^V12^-transformed cells to grow in reduced serum conditions and to avoid contact inhibition.

**Table 1:** Growth Curve Analysis (doubling time in hrs)

Cell Line	(+) Tet - GENE OFF	(-) Tet - GENE ON
MSU-1.1	30.2 (± 1.0)	29.8 (± 0.7)
PH3 MT	19.9 (± 0.5)	19.4 (± 0.7)
Vector Control	16.6 (± 0.5)	20.4 (± 1.2)*
Cdc42^N17^-C1	17.5 (± 2.0)	28.1 (± 2.0)**
Cdc42^N17^-C2	17.0 (± 1.7)	26.6 (± 1.2)**
Rac1^N17^-C1	16.3 (± 0.2)	31.2 (± 3.0)*
Rac1^N17^-C2	16.6 (± 0.7)	31.4 (± 1.0)**
Rac1^N17^/Cdc42^N17^	18.2 (± 0.5)	35.8 (± 1.2)*

Values represent Mean ± SD

*,** represent a significant difference compared to (-) Tet condition using a homoscedastic

two-tailed t-test (p < 0.05, p < 0.01, respectively), N = 3 for all conditions

**Figure 2:**
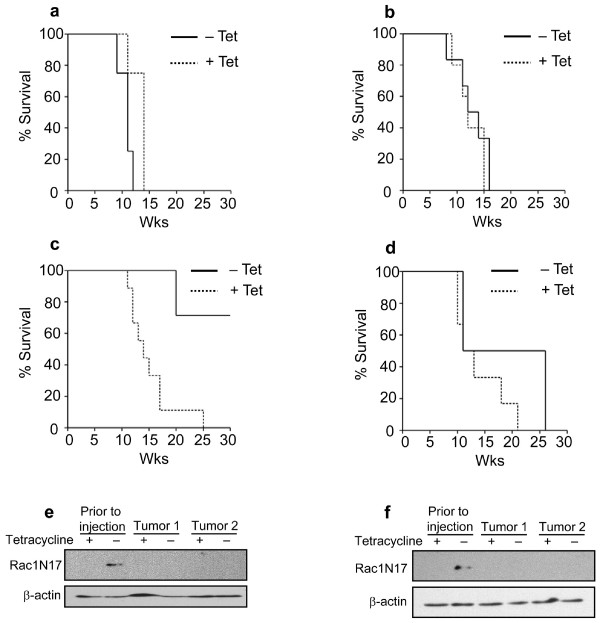
**Evidence that Rac1 activity is essential to maintain HRas^V12^-induced tumor formation**. Solid lines represent mice that were not administered tetracycline (Tet). Dotted lines represent mice injected with the same cell strain, but mice were given Tet in order to suppress dominant-negative expression. Tumors were measured weekly. When tumors reached a volume of approximately 0.5 cm^3^, mice were sacrificed and the tumors were removed for further analysis. The data are plotted using Kaplan-Meier analyses. (a) PH3MT-tTak-C1 (parent); N = 4 (- Tet), N = 4 (+ Tet) p > 0.1. (b) PH3MT-VC-C2 (vector control); N = 5 (- Tet), N = 6 (+ Tet) p > 0.1. (c) PH3MT-Rac1^N17^-C1; N = 7 (- Tet), N = 9 (+ Tet), p < 0.001. (d) PH3MT-Rac1^N17^-C2; N = 4 (- Tet), N = 6 (+ Tet), p > 0.1. (e & f) Western blots probed with myc (9E10) antibody to detect dominant-negative protein expression in two tumor-derived cell lines (Tumor 1 and Tumor 2) Both blots were probed with β-actin to verify loading.

**Figure 3:**
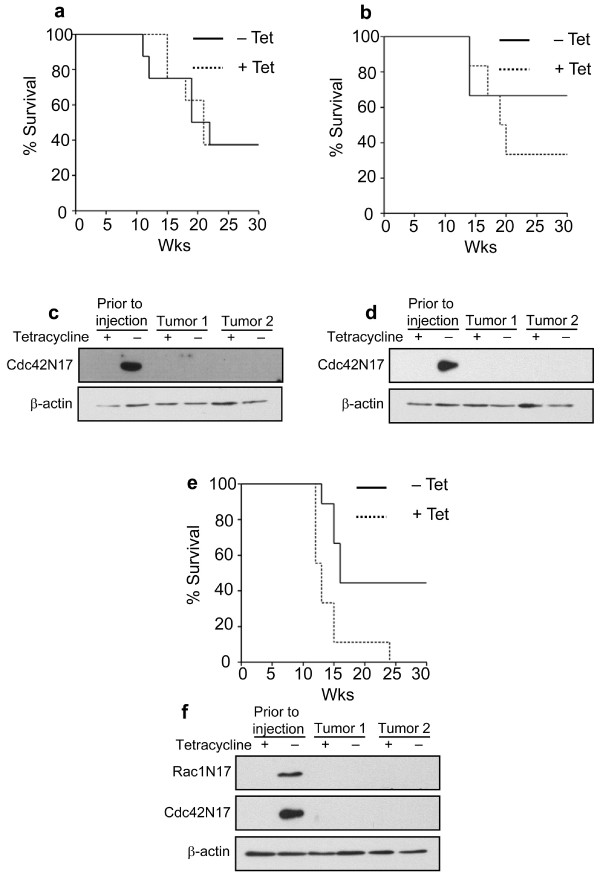
**Evidence that Rac1 and/or Cdc42 activity is essential to maintain HRas^V12^-induced tumor formation**. Solid lines represent mice that were not administered tetracycline (Tet). Dotted lines represent mice injected with the same cell strain, but mice were given Tet in order to suppress dominant-negative expression. Tumors were measured weekly. When tumors reached a volume of approximately 0.5 cm^3^, mice were sacrificed and the tumors were removed for study. The data are plotted using Kaplan-Meier analyses. (a) PH3MT-Cdc42^N17^-C1; N = 8 (- Tet), N = 8 (+ Tet), p > 0.1. (b) PH3MT-Cdc42^N17^-C2; N = 6 (- Tet), N = 6 (+ Tet), p > 0.1. (c & d) Western blots probed with FLAG antibody to detect dominant-negative protein expression in two tumor derived cell lines (Tumor 1 and Tumor 2). (e) PH3MT-Rac1^N17^/Cdc42^N17^; N = 9 (- Tet), N = 9 (+ Tet) p < 0.001. (f) Western blots probed with either myc (9E10) or FLAG antibody to detect dominant-negative protein expression in two tumor derived cell lines (Tumor 1 and Tumor 2). All blots were probed with β-actin to verify loading.

To determine whether the activities of Rac1 and/or Cdc42 proteins are required for maintaining the ability of PH3MT cells to form tumors in athymic mice, cells expressing Rac1^N17^, Cdc42^N17^, or both mutant proteins, were injected subcutaneously into athymic mice and tumor growth was monitored. As a control, another set of mice was injected with the parental cell strain, PH3MT-tTAk-C1, or with vector control cell strain PH3MT-VC-C2. By 15 weeks, all mice that had been injected with the latter two cell strains, in the presence or absence of tetracycline, developed tumors and were sacrificed (Fig. 2a &2b). In contrast, dominant-negative interference with Rac1 activity resulted in decreased tumor-forming ability, and mice injected with these cells showed a significant prolongation of a tumor-free lifespan (p < 0.01) (Fig. 2c &2d). However, not all mice escaped tumor formation. We hypothesized that following injection, cells with levels of dominant negative protein expression sufficient to prevent tumor growth were selected against, while cells with low levels of mutant protein expression were able to proliferate and resulted in tumor formation. To examine this possibility, we derived cell lines from resected tumors, selected for injected cells using the puromycin resistance marker, and compared the level of Rac1^N17 ^expression relative to the level expressed in the cell strain prior to injection. We were unable to detect Rac1^N17 ^in cell strains derived from these tumors (Fig. 2e &2f). These results strongly suggest that Rac1 activity plays a critical role in the ability of the transformed fibroblasts to form sarcomas, and that when the dominant-negative protein expression is suppressed, these cells re-acquire the ability to form such tumors. Although there was no significant difference in the length of survival of mice injected with cells expressing Cdc42^N17^, subsequent Western blotting of tumor-derived cell strains revealed similar results, i.e., these cell strains had lost detectable levels of expression of dominant-negative proteins (Fig. 3a-d).

Subcutaneous injection of PH3MT cells expressing both Rac1^N17 ^and Cdc42^N17 ^proteins resulted in significantly prolonged tumor-free survival (p < 0.0001) (Fig. 3e). Again, not all mice escaped tumor formation. Expression analysis of two tumor-derived cell lines revealed undetectable levels of dominant-negative protein expression (Fig. 3f). These data suggest that the activity of both Rac1 and Cdc42 is essential for HRas^V12^-induced transformation of human fibroblasts.

### Role of Rac1^V12 ^or Cdc42^V12 ^protein in the transformation of human fibroblasts

**Figure 4:**
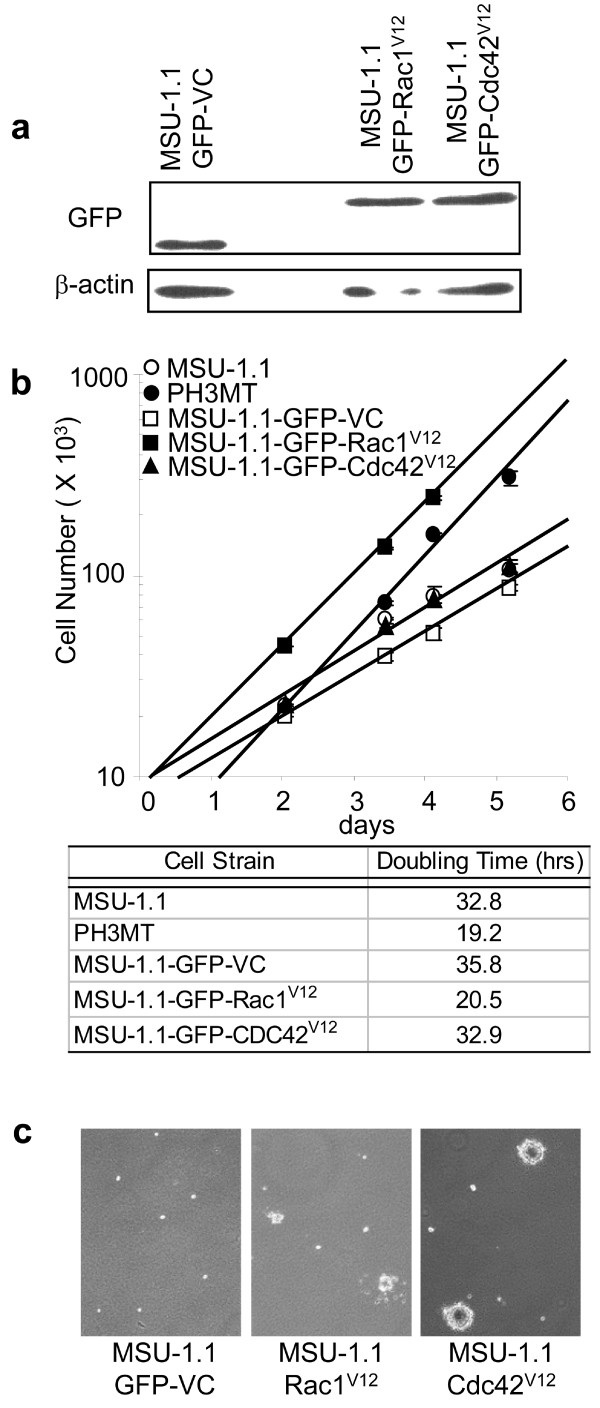
**Transformed phenotypes elicited by expression of Rac1^V12 ^or Cdc42^V12 ^in human fibroblasts**. (a) MSU-1.1 fibroblasts expressing either GFP alone (MSU-1.1-GFP-VC), or GFP-tagged constitutively-activated proteins. (b) The indicated cell strains were grown in medium with reduced serum (0.5% SCS). Growth curves were plotted. Doubling time was calculated based on an equation derived from a best-fit exponential curve when cells were in log-phase growth. Error bars represent the SD of triplicate experiments. (c) The indicated cell strains were plated in 0.33% agarose and grown for three weeks. Each picture represents one representative field from each cell line. This experiment was completed in triplicate, each with similar results.

To further address the roles of Rac1 and Cdc42 in the development of a transformed phenotype, we also evaluated the ability of cells expressing activated mutants to display transformed properties. Based on studies in rodent fibroblasts, we hypothesized that expression of Rac1^V12 ^or Cdc42^V12 ^in human fibroblasts would elicit a transformed phenotype [8,9]. To test this hypothesis, we stably transfected the parental MSU-1.1 cell strain with vectors encoding GFP-Rac1^V12 ^or GFP-Cdc42^V12 ^proteins (Fig. 4a), and assayed them for the ability to grow in medium with reduced serum, form large colonies in agarose, and develop into sarcomas in athymic mice. Expression of Rac1^V12^, but not Cdc42^V12 ^resulted in an increased ability of MSU-1.1 fibroblasts to grow in medium with reduced serum (Fig. 4b). Logarithmic extrapolation revealed a doubling time of 19 hr in HRas^V12 ^expressing PH3MT cells and 20 hr in MSU-1.1 cells expressing Rac1^V12^. This is in contrast to parental, vector-control, and Cdc42^V12 ^expressing cells which displayed doubling times of >30 hr.

The ability of cells to grow in reduced serum conditions typically correlates with the potential to form anchorage independent colonies in soft agar. To explore this possibility, these same cell strains were suspended in agarose and monitored for colony formation (Fig. 4c). We found that expression of Cdc42^V12 ^confers the ability for these cells to form large anchorage independent colonies, whereas expression of Rac1^V12 ^resulted in inconsistent small colony formation. Together, these data support the results of previous studies in mouse and rat fibroblasts indicating a role for Rac1 in proliferation in reduced serum conditions and Cdc42 in anchorage independent growth [8].

To determine whether Rac1^V12 ^or Cdc42^V12 ^expression in human fibroblasts results in malignant transformation, we subcutaneously injected these mutant expressing cell strains into the flanks of athymic mice. Surprisingly, neither Rac1^V12 ^nor Cdc42^V12 ^expression resulted in the ability for these cells to form tumors 28 weeks post injection (data not shown).

### Identification of Rac1 and Cdc42 regulated genes in HRas^V12^-transformed human fibroblasts

Because the activities of both Rac1 and Cdc42 play essential roles in mediating HRas^V12^-induced transformation, we hypothesized that downstream effectors of these two G-proteins play similarly indispensable roles. To identify these genes, mRNA was harvested from the PH3MT-Rac1^N17^/Cdc42^N17 ^cell strain grown either in the presence, or absence of tetracycline. Therefore, in this analysis, the same cell strain was used as both the control, i.e. normal Rac1 and Cdc42 signalling, and the experimental, i.e. inhibited Rac1 and Cdc42 signalling, groups. Using Affymetrix GeneChip technology, a total of 29 significant expression differences were identified (Table 2). The relatively small number and magnitude of significant gene changes identified in this analysis is likely attributable to the unique ability to compare transcriptome differences in the same cell line, thereby removing background "noise" typically observed in array analyses comparing transcriptome profiles in independent cell clones. Our criteria for choosing genes for follow-up experiments included those genes that: 1) were known to be affected by HRas mutations, and 2) had a known role in cancer. Two such genes, *uPA (urokinase plasminogen activator) *and *VEGF (vascular endothelial growth factor) *were of particular interest since previous work in our lab, and others, has shown that HRas transformed cells including PH3MT, as well as N-Ras and K-Ras transformed cells derived from the parental MSU-1.1 cell strain, secrete significantly elevated levels of both uPA and VEGF proteins compared to their non-transformed parental cell strain ([18,19] and data not shown). In the present study, our array analyses detected >1.5 fold decrease in both *VEGF *and *uPA *gene expression when both Rac1 and Cdc42 were inhibited, suggesting that Rac1 and/or Cdc42 play significant roles in uPA and VEGF expression downstream of oncogenic HRas.

**Table 2:** Summary of gene changes controlled by Rac1 and by Cdc42

*Genes up-regulated*		
Name	Fold Change	Reference

Ubiquitin Conjugating enzyme 12 (UBC12) *	6.4	[39]
Elongation factor-1 alpha-2 *	2.3	[40]
Guanine nucleotide-binding regulatory protein (G-y-alpha)	2.2	
mSin3A associated polypeptide p30 *	2.1	[41]
Rac protein kinase alpha	1.8	
Coupling protein G(s) alpha-subunit (alpha-S1)	1.8	
Histone H1x	1.8	
80K-L protein *	1.6	[42]
Killer cell lectin-like receptor, Subfam. C, member 2 (NKG2C) *	1.6	[43]
Alpha subunit of GsGTP binding protein	1.5	
Lipoprotein-associated coagulation inhibitor (LACI)	1.5	
ATP synthase alpha subunit	1.3	
Heat shock protein 70 (hsp70) *	1.3	[44]

***Genes down-regulated***		

Name	Fold Change	

Insulin-like growth factor-binding protein-3 *	-1.9	[45]
28s Ribosomal RNA gene	-1.6	
Cyclooxygenase-2 (hCox-2) *	-1.6	[46]
Vascular endothelial growth factor (VEGF) *	-1.6	[47]
Urokinase plasminogen activator (uPA) *	-1.5	[48]
Human KIAA0628	-1.5	
Asparagine synthetase	-1.5	
Axl tyrosine kinase receptor *	-1.5	[49-51]
High mobility group isoform C (HMGI-C) *	-1.5	[52-54]
Lnk adaptor protein	-1.4	
GTPase-activating protein ras p21 (RASA)	-1.4	
Human KIAA0728 protein	-1.3	
Axl tyrosine kinase receptor splice 2 *	-1.3	[49-51]
N-myristoyltransferase 2	-1.3	
Caveolin 2 *	-1.3	[55]
Glycosylphosphatidylinositol-H (GPI-H)	-1.3	

### Rac1 and Cdc42 independently regulate secreted levels of uPA protein in the context of activated HRas

**Figure 5:**
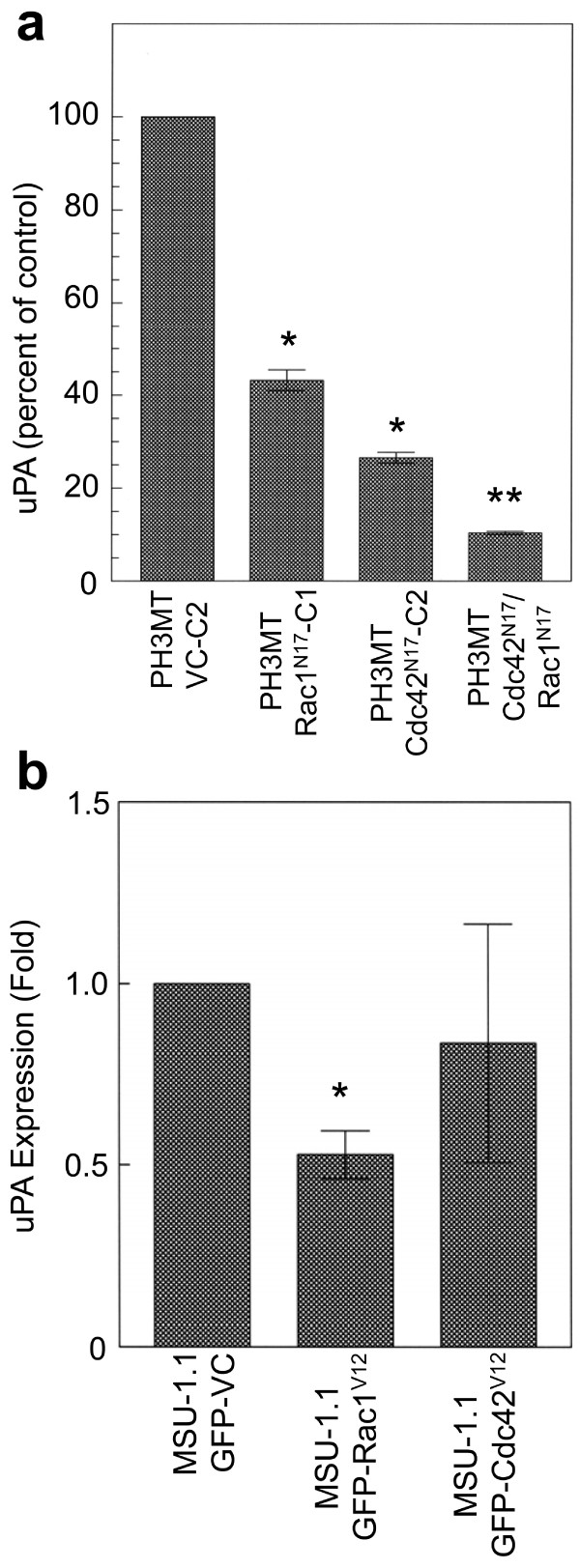
**Activated Rac1 and Cdc42 independently regulate uPA expression**. The indicated cell strains were serum starved for 24 hours then stimulated with medium containing 10% SCS. (a) Grown in the absence of tetracycline, PH3MT cell strains expressing a vector control (PH3MT-VC-C2), Rac1^N17^(PH3MT- Rac1^N17^-C1), Cdc42^N17 ^(PH3MT-Cdc42^N17^-C2) or both Rac1^N17 ^and Cdc42^N17 ^(PH3MT-Rac1^N17^/Cdc42^N17^) were tested for uPA expression levels using ELISA. Data is presented as percent of control. Error bars indicate the SD from triplicate experiments. * indicates significant difference, p < 0.05. ** indicates a significant difference compared to PH3MT-Rac1^N17^-C1 and PH3MT-Cdc42^N17^-C2, p < 0.05. (b) conditioned medium was collected from MSU-1.1 cells expressing GFP alone (MSU-1.1-GFP-VC), GFP-tagged Rac1^V12 ^(MSU-1.1-GFP-Rac1^V12^) or GFP-tagged Cdc42^V12 ^(MSU-1.1-GFP-Cdc42^V12^), and uPA expression was analyzed. Data is presented as fold-induction of uPA expression. Error bars represent the SD from triplicate experiments. * indicates significant difference, p < 0.05.

To determine whether the difference in *uPA *mRNA levels corresponded to alterations in secreted levels of uPA protein, ELISA analyses were conducted using tissue culture supernatants (Fig. 5a). Inhibition of either Rac1 or Cdc42 in PH3MT cells resulted in a 60% and 70% reduction in secreted uPA protein levels, respectively. Interestingly, inhibition of both proteins resulted in an additive reduction. Although our transcriptome array analyses did not indicate whether or both Rac1 and/or Cdc42 affected *uPA *gene expression, this analysis indicates that the activation of both proteins is required for the full elaboration of increased uPA secretion.

In a parallel experiment, we also determined whether expression of Rac1^V12 ^or Cdc42^V12 ^could increase the levels of secreted uPA protein in the parental, non-transformed MSU-1.1 cell strain (Fig. 5b). Interestingly, expression of neither Cdc42^V12 ^nor Rac1^V12 ^resulted in increased levels of secreted uPA protein, indicating that although their activities are required to mediate the secretion of uPA in Ras^V12^-transformed PH3MT cells, their activation alone is not sufficient to induce similar increases in expression. In fact, activation of Rac1 resulted in a small, but reproducible decrease in levels of secreted uPA protein.

### Rac1 and Cdc42 independently regulate secreted levels of VEGF protein

**Figure 6:**
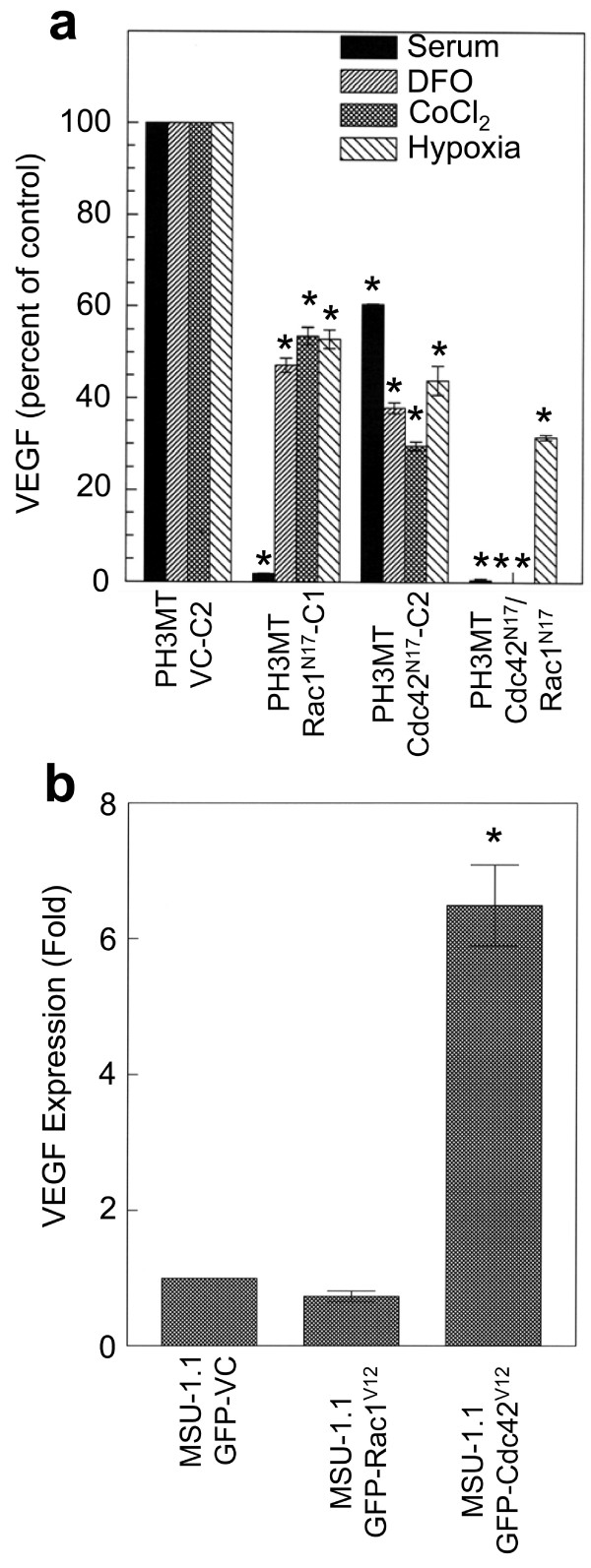
**Both Rac1 and Cdc42 regulate VEGF expression in non-hypoxic and hypoxic conditions**. All cell strains were serum starved for 24 hours prior to exposure to the indicated agent. Conditioned media were collected after 24 hr and ELISA analyses were completed. The data is presented as either percent of control, or fold induction as indicated in the figure. Error bars represent the SD from triplicate experiments. (a) PH3MT cell strains expressing a vector control (PH3MT-VC-C2), Rac1^N17 ^(PH3MT- Rac1^N17^-C1), Cdc42^N17 ^(PH3MT-Cdc42^N17^-C2), or both (PH3MT-Rac1^N17^/Cdc42^N17^), were stimulated with either medium containing 10% SCS, 100 μM DFO, 100 μM CoCl_2 _or Hypoxia (1% O_2_). (b) MSU-1.1 cells expressing GFP alone (MSU-1.1-GFP-VC-C2), or GFP-tagged Rac1^V12 ^(MSU-1.1-GFP-Rac1^V12^), or GFP-tagged Cdc42^V12 ^(MSU-1.1-GFP-Cdc42^V12^). Cells were stimulated with medium containing 10% SCS. * denotes a significant difference (p < 0.01).

To determine if Rac1 and/or Cdc42 regulate HRas^V12^-induced VEGF secretion, we measured the amount of VEGF protein secreted from PH3MT cell strains stably expressing Rac1^N17 ^and/or Cdc42^N17 ^dominant-negative mutants. Inhibition of Rac1 alone, or both Rac1 and Cdc42, completely abrogated HRas^V12^-induced secreted VEGF levels (Fig. 6a). However, inhibition of Cdc42 alone resulted in only a 40% reduction. These data indicate that in addition to Cdc42-mediated pathways, other signalling networks, dependent on Rac1 activity, play significant roles in VEGF secretion in the context of HRas^V12 ^activation.

Current studies indicate that both Rac1 and Cdc42 regulate hypoxia inducible factor (HIF)-induced VEGF expression in response to hypoxia in human hepatocellular carcinoma and gastric cancer cell lines [20,21]. For this reason, we hypothesized that both Rac1 and Cdc42 regulate secreted levels of VEGF protein from HRas^V12^-transformed fibroblasts grown in hypoxic conditions. To address this hypothesis, we cultured PH3MT derivative cell strains expressing Rac1^N17 ^and/or Cdc42^N17 ^under hypoxic (1% O_2_) conditions. Furthermore, we carried out parallel studies using two hypoxia mimetics, cobalt chloride (CoCl_2_) or deferoxamine (DFO). Under each condition, inhibition of either protein resulted in a 50% - 60% reduction in the level of secreted VEGF protein, whereas when these cells were exposed to either CoCl_2 _or DFO, inhibition of both Rac1 and Cdc42 completely eliminated detectable levels of VEGF protein (Fig. 6a). However, there was not an additive decrease in VEGF secretion in cells exposed to hypoxia. Therefore, these data indicate that both Rac1 and Cdc42 pathways are required for VEGF expression in HRas^V12^-transformed human fibroblasts exposed to hypoxic conditions.

Because the activation of Rac1 and Cdc42 is required for the full elaboration of VEGF expression downstream of oncogenic HRas, we hypothesized that introduction of Rac1^V12 ^or Cdc42^V12 ^protein in MSU-1.1 cells results in an induction of VEGF protein secretion (Fig. 6b). However, we found that expression of Rac1^V12 ^did not induce VEGF expression in human fibroblasts. In contrast, expression of Cdc42^V12 ^induced a significant increase in expression of VEGF (6-fold; p < 0.05). This suggests that in human fibroblasts, Rac1 regulates the levels of secreted VEGF in the context of HRas^V12^-induced mitogenic signalling, whereas the activation of Cdc42 is sufficient to increase levels of secreted VEGF, independent of other oncogenic HRas-mediated effector pathways.

## Discussion

In agreement with previous reports conducted in rodent fibroblast cell lines, the data obtained in this study show that both Rac1 and Cdc42 activity are essential for maintaining HRas^V12^-induced transformed phenotypes of human fibroblasts [8,9,22]. In addition, we found that activation of Rac1 in non-transformed human fibroblasts results in their ability to grow in the absence of serum-provided growth factors, and that activated Cdc42 potentiates anchorage independent growth. These data corroborate the results of studies conducted with rodent fibroblasts exhibiting these same properties, under identical physiological conditions, and with expression of identical mutant proteins [8,10]. A known role for Rho GTPase family members in general, and a specific role for both Rac1 and Cdc42 in various mitotic processes is but one possible way that inhibition of Rac1 and/or Cdc42 may affect the transformed phenotypes measured in this study [23]. In contrast to the results of rodent studies, expression of activated mutants in human MSU-1.1 cells failed to induce malignant transformation, as defined by tumor growth. It is possible that the GFP tag interferes with the function of these activated mutants, or that levels of these proteins were insufficient to fully transform the human cells. However, expression of these mutants did result in the manifestation of previously reported transformed properties. Therefore, it is likely that malignant transformation of human fibroblasts requires the simultaneous dysregulation of a wide array of independent signalling networks, in addition to those mediated by Rac1 and Cdc42. In support of these findings, recent reports indicate that modification of Ral and caveolin-1-mediated pathways are required for HRas^V12^-induced malignant transformation of human fibroblasts, but do not appear to be necessary for malignant transformation of rodent fibroblasts [11,24,25].

Because Rac1 and Cdc42 are essential mediators of HRas^V12^-induced transformation, we carried out Affymetrix GeneChip analyses of HRas^V12^-transformed cells expressing tetracycline-regulated Rac1^N17 ^and Cdc42^N17 ^dominant-negative proteins. This allowed us to use the same cell strain as the control and the experimental cell populations. This analysis, allowed us to identify a highly significant group of 29 genes whose level of expression was significantly mediated by Rac1 and/or Cdc42 activity, only 14 of which have previously been reported to play a role in cancer.

It is likely that both Rac1 and Cdc42 mediate the expression of downstream effectors by modulating both post-transcriptional and transcriptional mechanisms. For example, it is known that Ras regulates the activation of JNK pathway mediators which, in some instances, affect the stability of mRNA via A/U rich sequences in the 3'UTR of targeted gene products. Therefore, Rac1 and Cdc42 may regulate both uPA and VEGF expression by altering JNK activity, and thereby regulating uPA and VEGF mRNA stability [26,27].

Transcriptional mechanisms also regulate expression of the urokinase plasminogen activator (uPA) protein. Cooperation of two PEA3/AP-1 binding sites in the *uPA *promoter is required to mediate TPA-induced uPA expression in NIH3T3 fibroblasts [28]. Furthermore, it has recently been shown that activation of the PI3K/Akt pathway, known to be upstream of Rac1 and Cdc42 activity, positively correlates with uPA expression in an ovarian cancer cell line [29]. Research from our laboratory has demonstrated that receptor bound uPA is present at higher levels in fibroblasts capable of forming fibrosarcomas including those transformed by N-, K-, or HRas^V12 ^than it is in a number of normal, non-tumorigenic human fibroblast cell strains [18]. The results of our present study indicate that both Rac1 and Cdc42 regulate HRas^V12^-induced uPA expression in parallel pathways, perhaps through JNK and AP-1 regulation. However, expression of activated Rac1 or Cdc42 did not induce uPA protein expression in the parental MSU-1.1 human fibroblasts. This may be partially explained by the results reported by Aguirre-Ghiso et al. [30] who found that expression of dominant-negative RalA protein in v-Ras transformed NIH3T3 cells completely abrogated v-Ras-induced uPA expression, as well as blocked tumor formation. Taken together, these results strongly suggest that Ras-induced RalA, Rac1 and Cdc42 activities are all required to co-ordinately regulate secreted levels of uPA protein in human fibroblasts, and that expression of these proteins individually is not sufficient to induce uPA expression.

VEGF is the best-studied mediator of angiogenesis [31]. Our results show that in non-hypoxic conditions, HRas^V12 ^induces VEGF expression in a Rac1-dependent manner, whereas Cdc42 plays only a partial role. Therefore, it is tempting to postulate that in this instance, Cdc42 mediates one of multiple signalling pathways downstream of Rac1, that when inactivated, partially disrupts VEGF secretion. Ras-induced VEGF expression is regulated by multiple mechanisms, including increased transcription, translation and mRNA stabilization [20,27,32-34]. For example, it has been shown that activation of ERK1/2, through the Raf-mediated Ras pathway, increases the activity of HIF1 and Sp1 transcription factors [20,33]. However, recent data from our laboratory indicate that reduction of Sp1 expression in HRas^V12^-transformed human fibroblasts does not affect expression of VEGF [35]. Saniger et al. [34] recently reported that activation of either Rac1 or Cdc42 can induce transcription of VEGF through activation of JNK, which results in increased transcription of the VEGF gene product. Although multiple Rac1 and Cdc42 independent pathways affecting HIF1 transcriptional networks exist [36], it is possible that in HRas^V12^-transformed human fibroblasts, Rac1 may play a partial role in stabilizing HIF1 and/or activating JNK, which then modulates secretion of VEGF protein.

Rac1 and Cdc42 have also been implicated in hypoxic-induction of VEGF expression through regulation of p53 and VHL protein levels, as well as HIF1, and JNK activation [20,21,34]. In agreement with these studies, we find that both Rac1 and Cdc42 play a role in hypoxia-induced VEGF expression in HRas^V12^-transformed fibroblasts. We also show that both Rac1 and Cdc42 mediate VEGF expression under hypoxic conditions. However, under the same conditions, inhibition of both Rac1 and Cdc42 did not result in an additive suppression of VEGF. In contrast, complete reduction in VEGF secretion by these cells in the presence of hypoxia mimetics DFO and CoCl_2 _was observed. This may result from mechanistic differences in simulation of hypoxia using mimetics compared to true hypoxic conditions, a result that has been observed previously [20].

As noted above, In MSU-1.1 human cells, expression of activated Cdc42, but not activated Rac1, results in high levels of secreted VEGF protein. This indicates that Cdc42 regulates the expression of VEGF in the absence of other Ras-mediated effector pathways, whereas Rac1 does not. It has been shown that activation of Ost, the RhoA- and Cdc42-specific GEF, can potently induce JNK transcriptional pathways, and that Cdc42 can regulate EGFR signalling in an autocrine fashion, both potentially impacting on the expression of VEGF [37,38]. This may explain the ability of activated Cdc42, but not Rac1 to induce VEGF secretion in the absence of Ras activity.

## Conclusions

Our research provides an incremental advancement of what is currently known about the roles of Rac1 and Cdc42 in Ras-induced transformation. We demonstrate that although expression of constitutively-activated Rac1 and Cdc42 does not induce malignant-transformation of human fibroblasts, both Rac1 and Cdc42 are essential mediators of HRas^V12^-induced transformation of such cells. We also identify 29 Ras-induced Rac1- and Cdc42-regulated genes. Furthermore, we validate the roles of both Rac1 and Cdc42 in mediating the expression of both uPA and VEGF protein. Finally, we identify a role for both Rac1 and Cdc42 in the regulation of VEGF protein expression in conditions of hypoxia.

## Competing interests

The authors declare that they have no competing interests.

## Authors' contributions

DMA and K-HTD collected and analyzed data from all experiments and co-authored the manuscript. SOR completed all animal experiments. VMM extensively reviewed and edited the manuscript. JJM supported and directed the study. All authors reviewed and approved the final manuscript.

## Pre-publication history

The pre-publication history for this paper can be accessed here:

http://www.biomedcentral.com/1471-2407/10/13/prepub
